# Maintenance of the Self-Reported Behavior Change Stage Among Male Bus Drivers Using Longitudinal Occupational Health Checkup Data: Retrospective Cohort Study

**DOI:** 10.2196/93645

**Published:** 2026-09-01

**Authors:** Eiko Nakanishi, Reiko Sakashita, Masakazu Morimoto, Manabu Nii

**Affiliations:** 1 College of Nursing Art and Science, University of Hyogo, 13-71 Kitaoji-cho, Akashi, Hyōgo, 6738588, Japan, 81 789259410, 81 789250878; 2 Graduate School of Engineering, University of Hyogo, Himeji, Hyōgo, Japan

**Keywords:** bus drivers, occupational health, health checkups, behavior change, self-reported behavior change stage, lifestyle, physical activity, smoking, metabolic syndrome, retrospective cohort study

## Abstract

**Background:**

Public bus drivers experience prolonged sitting, irregular shifts, limited breaks, and disrupted meals and sleep, making the maintenance of a healthy lifestyle difficult. Although occupational health checkups identify health risks, little is known about the duration of maintenance of self-reported stages of behavior change or their association with physiological markers.

**Objective:**

This study examined the duration of maintenance of the self-reported stage of behavior change and its association with physiological markers and lifestyle behaviors among male bus drivers.

**Methods:**

This retrospective cohort study analyzed occupational health checkup data collected from male bus drivers at a passenger bus company in Japan between 2006 and 2023. The primary exposure was the self-reported behavior change stage, based on a standard questionnaire item used in the Specific Health Checkups questionnaire. For the duration analysis, each participant’s longest continuous action or maintenance episode was identified, and the Kaplan-Meier analysis was used to estimate the retention time. For the outcome analyses, participants who maintained the action or maintenance stage for at least 1.5 years after time 0 were classified as the sustained group. Those who returned to the preaction or preparatory stage at 6 months or 1 year comprised the comparison group. Physiological markers were compared using analysis of covariance adjusted for baseline values, age, and medication use. Lifestyle items were analyzed using multivariable logistic regression.

**Results:**

The survival analysis dataset included 717 participants, of whom 566 (78.9%) returned to the preaction or preparatory stage, while 151 (21.1%) were right-censored. The mean age at the start of the maintenance episode was 47.2 (SD 7.0) years, and the median retention time was 362 (IQR 0-1087) days. Retention probabilities were 57.7% at 6 months, 44.1% at 1 year, 33.3% at 2 years, and 23.2% at 3 years. In the outcome analyses, the sustained and comparison groups included 249 and 468 participants, respectively. At baseline, waist circumference was lower in the sustained group (85.77 cm vs 87.54 cm; *P*=.02), whereas other markers and medication use did not differ significantly between groups. At 2 and 3 years, the sustained group had lower BMI, waist circumference, triglycerides, and higher high-density lipoprotein cholesterol levels. Glycated hemoglobin (HbA_1c_) levels were lower in the sustained group at 3 years (*P*=.02). The sustained group had higher odds of an exercise habit (1 year: odds ratio [OR] 3.40, 95% CI 2.24-5.16; 2 years: OR 8.90, 95% CI 5.26-15.07) and daily physical activity (1 year: OR 2.51, 95% CI 1.61-3.91; 2 years: OR 5.17, 95% CI 2.93-9.13), and lower odds of current smoking (1 year: OR 0.36, 95% CI 0.14-0.95; 3 years: OR 0.26, 95% CI 0.09-0.75).

**Conclusions:**

Maintenance of the self-reported behavior change stage often began in midlife but was difficult to sustain beyond 1 year. Long-term maintenance was associated with favorable physical activity–related lifestyle patterns, lower smoking prevalence, and more favorable metabolic profiles. Health guidance should evaluate not only numerical improvements but also sustained behaviors and the prevention of worsening under occupational constraints.

## Introduction

### Background

Public bus transportation is an essential form of social infrastructure that supports mobility, participation in community activities, and access to health care, particularly in aging societies. However, the bus industry in Japan faces serious challenges, including an aging workforce and a shortage of drivers [1]. For bus operators, maintaining driver health is not merely an individual health issue but a prerequisite for sustaining safe and reliable public transportation services.

Bus drivers work under conditions characterized by prolonged sitting, continuous attention to road and passenger safety, irregular breaks, early-morning or late-night shifts, and limited flexibility in adjusting their eating, sleeping, and exercise habits. In response to these occupational conditions, regular health checkups are legally required at least once a year and once every 6 months for those engaged in late-night work. In addition, various screening examinations, such as brain checkups and screening for sleep apnea syndrome, are recommended to prevent serious accidents [2]. In daily operations, bus operators are required to conduct preduty and postduty roll calls to check drivers’ health status and to establish systems that encourage drivers to promptly report warning signs or symptoms of serious diseases, such as stroke [3].

However, for bus drivers working under demanding schedules, initiating and maintaining healthy behaviors is difficult, even when health risks are identified during regular health checkups. Disrupted lifestyle habits [3], high smoking rates [4], and prolonged sitting have been reported among bus drivers and may increase their risk of diabetes and cardiovascular disease compared with the risk among general workers [5,6]. In Japan, health-related accidents and duty interruptions among commercial motor vehicle drivers, including bus and taxi drivers, have more than doubled over the past decade and remain at a high level; heart disease, cerebrovascular disease, and aortic aneurysm or dissection account for approximately 30% of these events [7]. Because these conditions are closely linked to modifiable risk factors such as hypertension, diabetes, dyslipidemia, obesity, smoking, insufficient sleep, and physical inactivity, regular health management and sustained lifestyle improvement are important for both driver health and public safety [7].

In Japan, Specific Health Guidance is provided to support individuals at risk of developing metabolic syndrome and to prevent lifestyle-related diseases. Although Specific Health Guidance has been associated with improvements in body weight and glycemic control, its effects on systolic blood pressure (SBP) and low-density lipoprotein cholesterol (LDL-C) levels have not been consistent, indicating challenges in lifestyle disease prevention [8]. For occupational groups with irregular work schedules, the provision of health information may be insufficient. Even when drivers understand the importance of healthy behaviors, they may face substantial barriers to initiating and maintaining new habits over time. Therefore, occupational health and nursing interventions need to address not only risk awareness but also motivation, self-efficacy, feasibility within work schedules, and the perceived personal benefits of behavior change.

Behavior change is a dynamic process rather than a single event. The transtheoretical model distinguishes stages such as contemplation, preparation, action, and maintenance, and emphasizes that long-term maintenance is required for stable health improvement [9]. However, in high-stress or highly constrained work environments, even initiated behavior change may be difficult to sustain, leading to discontinuation or relapse into previous habits [10,11]. Digital and mobile health (mHealth) studies suggest that support incorporating psychological benefits, such as empowerment, improved well-being, and personalized feedback, may facilitate behavior change beyond information provision alone [12,13]. Therefore, effective health support for bus drivers should help translate health checkup results into feasible, meaningful, and sustainable actions within their occupational constraints.

Occupational health checkups provide repeated longitudinal data that enable evaluation of self-reported behavior change stages and physiological markers over time. However, limited evidence is available on how long the self-reported behavior change stage is maintained among occupational drivers or whether long-term maintenance is associated with measurable differences in physiological markers. Therefore, this study examined the duration of maintenance of the self-reported behavior change stage and the association between long-term maintenance and changes in physiological markers, such as glycated hemoglobin (HbA_1c_) and lipid profiles, using historical occupational health checkup data from male bus drivers. The findings may provide foundational evidence for developing occupational health and nursing support models that promote sustained behavior change among bus drivers.

### Study Objectives

This study aimed to examine the duration of maintenance of the self-reported behavior change stage and the association between long-term maintenance of this stage and changes in physiological markers using longitudinal occupational health checkup data from male bus drivers.

## Methods

### Study Design and Participants

This retrospective cohort study analyzed occupational health checkup data collected from male bus drivers employed by a general passenger bus company in Japan (hereafter referred to as Company A) between 2006 and 2023. The eligible participants were male employees who had undergone at least 2 health checkups during the study period. All bus drivers are required to undergo regular health checkups. Health checkups were conducted annually until 2013 and twice annually from 2014 onward. The final observation year was 2023, for which data were available for all eligible participants.

### Measures

We used anthropometric measurements obtained during mandatory health checkups, including waist circumference (WC), BMI, SBP, and diastolic blood pressure (DBP), and blood test results, including HbA_1c_, measured according to the National Glycohemoglobin Standardization Program (NGSP), high-density lipoprotein cholesterol (HDL-C), LDL-C, and triglycerides (TG). Lifestyle habits, including exercise, dietary habits, alcohol consumption, smoking, and sleep, were assessed using a standard Self-Administered Questionnaire (SAQ) for Specific Health Checkups provided by the Ministry of Health, Labour, and Welfare [14]. The detailed definitions of the questionnaire items are provided in Table S1 in Multimedia Appendix 1.

### Operational Definitions

#### Self-Reported Behavior Change Stage

The self-reported behavior change stage was operationally defined as the self-reported stage of behavior change based on the standard SAQ items used in the Specific Health Checkups questionnaire. The question was, “Do you intend to improve your lifestyle habits, such as eating and exercise habits?” The response options were as follows: (1) no intention to improve, (2) intend to improve within the next 6 months, (3) intend to improve within the next month and have already started taking action gradually, (4) already taking action for less than 6 months, and (5) already taking action for 6 months or more. In this study, responses (1) to (3) were classified as the preaction or preparatory stage and coded as a self-reported behavior change stage flag of 0, whereas responses (4) and (5) were classified as the action or maintenance stage and coded as a self-reported behavior change stage flag of 1 (Table S2 in Multimedia Appendix 1) [14]. This measure captures the self-reported stage of behavior change related to lifestyle improvement and does not objectively measure actual behavioral quantities, such as physical activity level or dietary intake.

#### At-Risk Status

At-risk status was defined as meeting at least 1 metabolic risk criterion in the Specific Health Checkups program: BMI≥25 kg/m² or WC≥85 cm; BP≥130/85 mm Hg; TG≥150 mg/dL or HDL-C<40 mg/dL; or HbA_1c_≥5.6% [15].

### Statistical Analysis

All analyses were performed using Python version 3.13.9. Statistical significance was assessed using 2-sided tests with α=.05.

### Duration of the Self-Reported Behavior Change Stage Maintenance

Participants who never met the criteria for at-risk status during the observation period were excluded. Next, participants who had never entered the action or maintenance stage of the self-reported behavior change stage were excluded.

The date of self-reported behavior change stage initiation was defined as the health checkup date on which a transition to the action or maintenance stage (self-reported behavior change stage flag=1) was confirmed. This date was defined as time 0, the starting point for survival analysis.

In this study, we analyzed the longest continuous maintenance episode observed for each participant during the observation period, rather than the first episode of the self-reported behavior change stage transition. This approach was chosen because, in occupational health checkup data, temporary transitions into and out of the action or maintenance stage may be observed repeatedly from a young age, and the first episode may not necessarily reflect clinically meaningful, sustained behavioral changes. The aim of this study was not to estimate the natural course after the first initiation of behavior change but to describe the duration of the most stable behavior change maintenance episode achieved by male bus drivers during the observation period and to examine the physiological and lifestyle characteristics associated with long-term maintenance. Therefore, for each participant, we identified the longest episode during which the action or maintenance stage was continuously observed; the start date of that episode was defined as time 0. The start date of the longest episode was used as the individual baseline to calculate the subsequent survival time, that is, the maintenance duration.

### Survival Analysis

To estimate the duration of the self-reported behavior change stage maintenance, the Kaplan-Meier method was used to estimate the median duration for which the action or maintenance stage was maintained from the start date defined above. Survival time was calculated from the start date to the health checkup at which discontinuation of the action or maintenance stage was first observed, or to the final health checkup for censored participants.

An event was defined as a transition from the action or maintenance stage (flag=1) to the preaction or preparatory stage (flag=0), indicating discontinuation of the action or maintenance stage. Participants who remained in the action or maintenance stage at their final observation in 2023 were right-censored. As self-reported behavioral transitions could be identified only at health checkup visits, the actual dates of initiation and discontinuation may have occurred within the interval between visits. As the health checkups were conducted annually until 2013 and semiannually thereafter, estimates of maintenance duration were subject to interval censoring.

### Outcomes Associated With the Sustained Self-Reported Behavior Change Stage

#### Definition of the Sustained and Comparison Groups

To examine the association between the long-term maintenance of the self-reported behavior change stage and physiological markers and lifestyle items, we constructed a pseudo-prospective cohort dataset based on the longest episode identified for each participant in “duration of self-reported behavior change stage maintenance” and followed participants’ health checkup data after time 0.

In the survival analysis for “duration of self-reported behavior change stage maintenance,” the median duration of continuous self-reported behavior change stage maintenance was approximately 1 year. Because regular health checkups were conducted approximately every 6 months from 2014 onward, self-reported behavior change stage maintenance status was observed at discrete follow-up points, such as 6 months, 1, 1.5, and 2 years after time 0. Therefore, the first observation point at which maintenance beyond the median duration was confirmed was 1.5 years. Accordingly, participants who remained in the action or maintenance stage continuously for at least 1.5 years after time 0 were operationally classified as the sustained group. Participants who transitioned to the preaction or preparatory stage at 6 months or 1 year after time 0 were classified as the comparison group. As the groups were defined retrospectively based on the duration of self-reported behavior change stage maintenance, group differences were interpreted as associations with long-term maintenance rather than causal effects.

#### Baseline Characteristics

To assess the comparability of baseline characteristics between the sustained and comparison groups in the constructed cohort dataset and to support the validity of subsequent outcome comparisons, participant characteristics at time 0 were compared between the groups.

For continuous variables, including age, WC, BMI, SBP, DBP, HbA_1c_, HDL-C, LDL-C, and TG levels, means and SDs were calculated, and between-group comparisons were performed using the 2-tailed Welch *t* test, which is robust to unequal variances and does not rely on the assumption of equal variances. For categorical variables, including the use of antihypertensive, antidiabetic, and lipid-lowering medications, frequencies and percentages were calculated, and between-group differences were assessed using the Pearson chi-square test.

#### Longitudinal Evaluation of Physiological Markers Using Analysis of Covariance

Between-group comparisons of physiological markers, including WC, BMI, SBP, DBP, HbA_1c_, HDL-C, LDL-C, and TG, were conducted at each follow-up point (6 mo, 1, 1.5, 2, 2.5, and 3 y). Analysis of covariance (ANCOVA) was used to adjust for the baseline values of each marker. The model was designed to account for baseline physiological differences between the groups and regression to the mean. Each follow-up physiological marker was entered as the dependent variable, and the group status—that is, the sustained group vs the comparison group—was entered as the main explanatory variable. Covariates included the baseline value of the corresponding marker, baseline age, and the use of antihypertensive, antidiabetic, and lipid-lowering medications at baseline and at the corresponding follow-up time points. This approach allowed us to estimate differences in physiological markers between the sustained and comparison groups while accounting for the influence of medication use to the extent possible.

### Association Between Lifestyle Items and Sustained Self-Reported Behavior Change Stage Using Logistic Regression

To explore lifestyle patterns that may be associated with differences in physiological markers observed in the sustained group, multivariable logistic regression analyses were conducted, using each of the 9 lifestyle items, including exercise habits, smoking status, and daily physical activity, as binary dependent variables coded as 1 for present or applicable and 0 for absent or not applicable.

Each logistic regression model included group status, baseline status of the corresponding lifestyle item, baseline age, and medication use at both baseline and each follow-up time point as covariates. Medications used included antihypertensive, antidiabetic, and lipid-lowering agents. We estimated odds ratios (ORs) and 95% CIs for the odds of each lifestyle item being present or applicable in the sustained group compared with the comparison group at each follow-up point. The 9 lifestyle items in this analysis were positioned for exploratory profiling to identify lifestyle patterns that may be associated with between-group differences in physiological markers accompanying long-term self-reported behavior change stage maintenance. Therefore, no uniform adjustment was applied for multiple comparisons.

### Ethical Considerations

The study was approved by the Research Ethics Committee of the Research Institute of Nursing Care for People and Community, University of Hyogo (approval number 2022F08). Informed consent was obtained from participants through the company’s internal bulletin board using an opt-out method. The employer irreversibly deidentified the dataset before transfer to ensure participant anonymity in accordance with Japanese privacy regulations.

## Results

### Duration of Self-Reported Behavior Change Stage Maintenance

The dataset constructed for survival analysis included 717 participants. Among them, 566 (78.9%) experienced an event that was defined as a transition back to the preaction or preparatory stage. The remaining 151 (21.1%) participants maintained the action or maintenance stage until the end of the follow-up and were treated as right-censored. At the start of the self-reported behavior change stage maintenance episode, that is, at baseline, the mean age of the participants was 47.2 (SD 7.0; range 25‐67) years.

**Figure 1.**
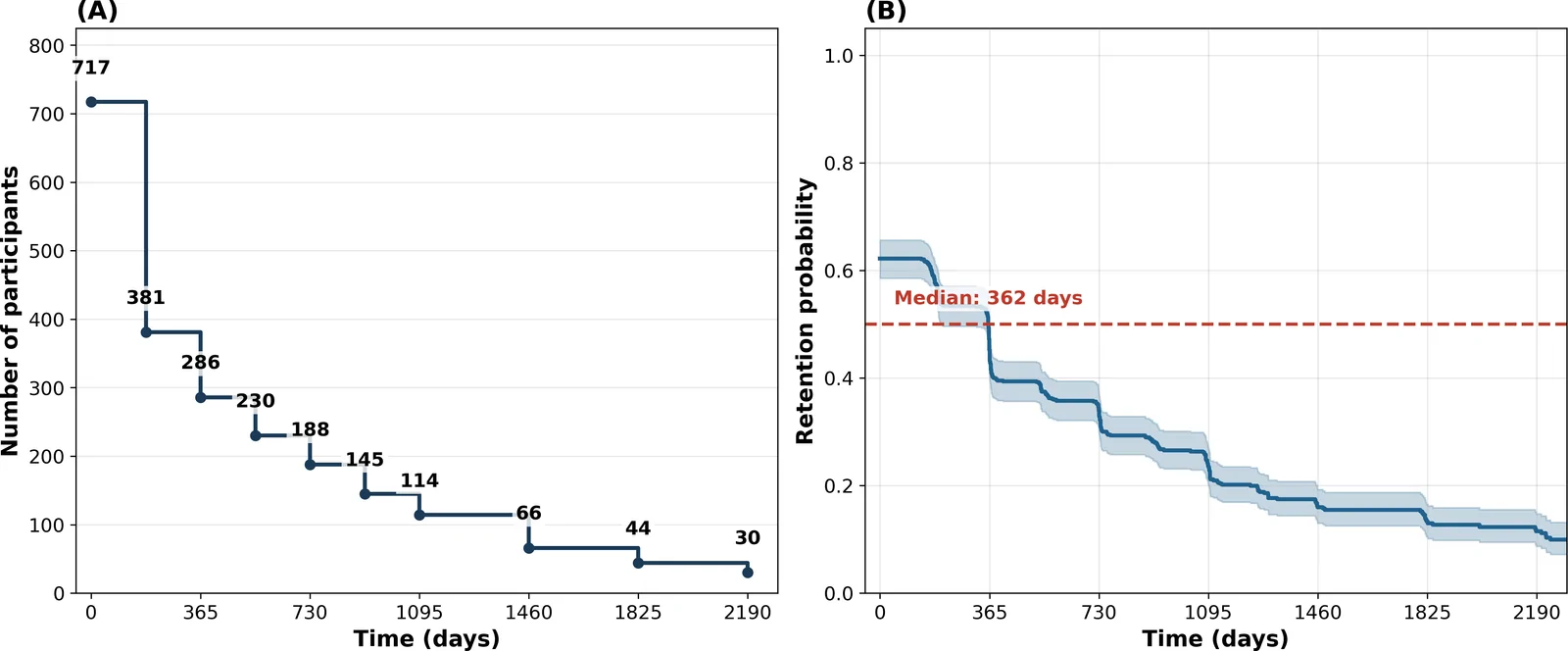
Duration of the self-reported maintenance of behavior change stage among male bus drivers. Panel A shows the number of participants continuing to maintain behavior change over time, and panel B shows the Kaplan-Meier retention curve.

Kaplan-Meier survival analysis showed that the median retention time for self-reported behavior change stage maintenance was 362 (IQR 0-1087) days. After the start of the action or maintenance stage, the retention probability was 57.7% (95% CI 54.0%‐61.2%) at 6 months (183 d) and continued to decline over time. Specifically, the retention probability was 44.1% (95% CI 40.3%‐47.8%) at 1 year (365 d), 33.3% (95% CI 29.6%‐36.9%) at 2 years (730 d), and 23.2% (95% CI 19.9%‐26.7%) at 3 years (1096 d; Figure 1).

### Outcomes Associated With the Sustained Self-Reported Behavior Change Stage

#### Baseline Characteristics

The baseline characteristics of the sustained (n=249) and comparison (n=468) groups at time 0 are shown in Table 1.

**Table 1.** Baseline characteristics of participants according to duration of self-reported behavior change stage maintenance^a^.

Item	Comparison (n=468)	Sustained (n=249)	Statistic (*df*)	*P* value
Demographic characteristic, mean (SD)				
Age (y)	47.19 (7.14)	47.24 (6.78)	−0.09 (529.3)	.93
Anthropometric parameters, mean (SD)				
BMI (kg/m²)	24.98 (4.01)	24.46 (3.50)	1.82 (568.1)	.07
WC^b^ (cm)	87.54 (10.74)	85.77 (9.16)	2.32 (578.6)	.02
Physiological parameters, mean (SD)				
SBP^c^ (mm Hg)	128.57 (12.84)	127.68 (12.39)	0.91 (521.8)	.36
DBP^d^ (mm Hg)	82.73 (9.88)	81.71 (9.44)	1.35 (526.0)	.18
HbA_1c_^e^ (%)	5.69 (0.60)	5.67 (0.54)	0.50 (549.6)	.62
TG^f^ (mg/dL)	131.04 (96.98)	125.76 (91.99)	0.72 (529.7)	.47
HDL-C^g^ (mg/dL)	58.95 (16.24)	59.44 (15.33)	−0.40 (531.8)	.69
LDL-C^h^ (mg/dL)	126.48 (28.52)	127.05 (28.16)	−0.26 (511.6)	.80
Medication use^i^, n (%)				
Antihypertensive	86 (18.4)	35 (14.1)	1.87 (1)	.17
Antidiabetic	32 (6.8)	13 (5.2)	0.47 (1)	.49
Lipid-lowering	69 (14.7)	29 (11.6)	1.07 (1)	.30

a Data were expressed as mean (SD) for continuous variables and n (%) for categorical variables. Test statistics were presented as *t* tests (*df*) for continuous variables and chi-square tests (*df*) for categorical variables. *P* values were calculated using the Welch *t* test for continuous variables and the Pearson chi-square test for categorical variables.

b WC: waist circumference.

c SBP: systolic blood pressure.

d DBP: diastolic blood pressure.

e HbA_1c_: glycated hemoglobin.

f TG: triglycerides.

g HDL-C: high-density lipoprotein cholesterol.

h LDL-C: low-density lipoprotein cholesterol.

i Medication use referred to the use of antihypertensive, antidiabetic, or lipid-lowering medications at baseline.

At baseline, WC was lower in the sustained group than in the comparison group (mean 85.77, SD 9.16 cm vs mean 87.54, SD 10.74 cm; *P*=.02). However, no statistically significant differences were observed between the groups with respect to age; other physiological markers, including BMI, BP, lipid markers, and glycemic status; or the proportion of participants using the 3 types of medications. These findings indicate that the clinical characteristics of the 2 groups at the start of the self-reported behavior change maintenance stage were generally comparable, except for WC.

#### Longitudinal Evaluation of Physiological Markers Using ANCOVA

Between-group comparisons of physiological markers at each follow-up point (6 mo, 1, 1.5, 2, 2.5, and 3 y) were conducted using ANCOVA, adjusting for the baseline value of each respective marker. To concisely present the long-term trajectory during the follow-up period, Table 2 shows the adjusted mean differences, 95% CIs, and *P* values at the main annual follow-up points of 1, 2, and 3 years. The adjusted means for both groups and the full results for all follow-up points, including 6 months, 1.5 years, and 2.5 years, are provided in Table S3 in Multimedia Appendix 1.

**Table 2.** Analysis of covariance-adjusted comparison of physiological markers over 3 years^a^.

Item	Comparison/sustained group, n	Difference (95% CI)	*P* value
Anthropometry			
BMI, kg/m^2^			
Year 1	390/247	−0.12 (−0.26 to 0.03)	.11
Year 2	299/233	−0.37 (−0.57 to −0.17)	<.001
Year 3	228/184	−0.36 (−0.61 to −0.10)	.006
WC^b^, cm			
Year 1	390/247	−0.28 (−0.80 to 0.24)	.29
Year 2	299/233	−1.27 (−1.93 to −0.60)	<.001
Year 3	228/184	−1.24 (−2.10 to −0.38)	.005
Blood pressure			
SBP^c^, mm Hg			
Year 1	390/247	−0.12 (−1.86 to 1.61)	.89
Year 2	299/233	1.21 (−0.81 to 3.22)	.24
Year 3	228/184	−0.56 (−2.79 to 1.67)	.62
DBP^d^, mm Hg			
Year 1	390/247	0.15 (−1.06 to 1.37)	.80
Year 2	299/233	0.10 (−1.27 to 1.47)	.89
Year 3	228/184	−0.87 (−2.43 to 0.69)	.28
Glucose and lipids			
HbA_1c_^e^, %			
Year 1	390/247	−0.01 (−0.07 to 0.05)	.74
Year 2	299/233	−0.03 (−0.08 to 0.01)	.17
Year 3	228/184	−0.07 (−0.12 to -0.01)	.02
TG^f^, mg/dL			
Year 1	390/247	6.44 (−6.63 to 19.51)	.33
Year 2	299/233	−17.03 (−30.51 to −3.56)	.01
Year 3	228/184	−21.22 (−37.28 to −5.15)	.01
HDL-C^g^, mg/dL			
Year 1	390/247	1.31 (0.11 to 2.50)	.03
Year 2	299/233	3.17 (1.80 to 4.53)	<.001
Year 3	228/184	3.69 (2.09 to 5.29)	<.001
LDL-C^h^, mg/dL			
Year 1	390/247	−0.58 (−3.71 to 2.56)	.72
Year 2	299/233	−0.40 (−4.22 to 3.42)	.84
Year 3	228/184	−0.55 (−4.61 to 3.51)	.79

a The difference indicates the adjusted mean difference, calculated as the difference between the sustained group and the comparison group. Negative values indicate lower adjusted means in the sustained group than in the comparison group. Differences, 95% CIs, and *P* values were calculated using analysis of covariance, adjusting for the baseline value of each marker, baseline age, and medication status for antihypertensive, antidiabetic, and lipid-lowering medications at both baseline and at each follow-up point.

b WC: waist circumference.

c SBP: systolic blood pressure.

d DBP: diastolic blood pressure.

e HbA_1c_: glycated hemoglobin.

f TG: triglycerides.

g HDL-C: high-density lipoprotein cholesterol.

h LDL-C: low-density lipoprotein cholesterol.

The sustained group had a lower BMI than the comparison group at 2 years (adjusted difference −0.37, 95% CI −0.57 to −0.17; *P*<.001) and 3 years (adjusted difference −0.36, 95% CI −0.61 to −0.10; *P*=.006). WC was also lower in the sustained group at 2 years (adjusted difference −1.27, 95% CI −1.93 to −0.60; *P*<.001) and 3 years (adjusted difference −1.24, 95% CI −2.10 to −0.38; *P*=.005). The sustained group maintained adjusted mean BMI values below the threshold for at-risk status. In contrast, the adjusted mean WC values in both groups remained above the predefined at-risk threshold of 85 cm throughout the follow-up period. At 3 years, the adjusted mean WC was 86.89 cm (SE 0.69 cm) in the sustained group and 88.13 cm (SE 0.67 cm) in the comparison group.

HDL-C values were higher in the sustained group at 1 year (adjusted difference 1.31, 95% CI 0.11-2.50; *P*=.03), 2 years (adjusted difference 3.17, 95% CI 1.80-4.53; *P*<.001), and 3 years (adjusted difference 3.69, 95% CI 2.09-5.29; *P*<.001). In addition, TG values were lower in the sustained group at 2 years (adjusted difference −17.03, 95% CI −30.51 to −3.56; *P*=.01) and 3 years (adjusted difference −21.22, 95% CI −37.28 to −5.15; *P*=.01). Regarding glycemic control, HbA_1c_ at 3 years was significantly lower in the sustained group than in the comparison group (*P*=.02). However, the adjusted mean HbA_1c_ values in both groups remained above the predefined at-risk status threshold of 5.6% throughout the follow-up period. At 3 years, the adjusted mean HbA_1c_ was 5.73% (SE 0.04) in the sustained group and 5.79% (SE 0.04) in the comparison group. No significant between-group differences were observed in SBP or DBP throughout the follow-up period.

### Association Between Lifestyle Items and Sustained Self-Reported Behavior Change Stage Using Logistic Regression

We constructed a cohort dataset using each participant’s maintenance episode of the self-reported behavior change stage, linked to health checkup data after time 0, to examine the association between long-term maintenance and physiological markers and lifestyle items.

As the groups were defined retrospectively based on the duration of self-reported behavior change stage maintenance, differences between the groups were interpreted as associations with long-term maintenance rather than causal effects. To explore lifestyle patterns that may be associated with between-group differences in physiological markers in the sustained group, multivariable logistic regression analyses were conducted using 9 lifestyle items as dependent variables. Table 3 shows the main lifestyle items that showed consistent between-group differences at 1, 2, and 3 years. The full results for all 9 lifestyle items and all follow-up points, including 6 months, 1.5 years, and 2.5 years, are provided in Table S4 in Multimedia Appendix 1.

In the exploratory analyses, the sustained group showed consistently higher odds of exercising and engaging in daily physical activity than the comparison group. The sustained group also showed significantly lower odds of current smoking at 1, 2, and 3 years.

Specifically, the prevalence of a regular exercise habit remained above 50% in the sustained group across the follow-up period (50.7%‐55.3%), whereas it was markedly lower in the comparison group (12.1%‐20.1%). Accordingly, the odds of having an exercise habit were significantly higher in the sustained group (OR range 3.40‐8.90; *P*<.001). Similarly, the prevalence of daily physical activity remained at approximately 30% in the sustained group (29.2%‐32.7%) but was lower in the comparison group (7.5%‐12.8%). The sustained group showed significantly higher odds of daily physical activity at all annual follow-up points (OR range 2.51‐5.17; *P*<.001). Regarding current smoking, the prevalence in the sustained group decreased from 25.5% at 1 year to 22.8% at 3 years, whereas it increased slightly from 31.5% to 32.5% in the comparison group.

In the adjusted logistic regression analyses, the sustained group had lower odds of current smoking at 1 year (OR 0.36, 95% CI 0.14-0.95; *P*=.04), 2 years (OR 0.32, 95% CI 0.12-0.87; *P*=.03), and 3 years (OR 0.26, 95% CI 0.09-0.75; *P*=.01). In addition, a higher proportion of participants in the comparison group (67.1%‐67.7%) than in the sustained group (46.2%‐53.3%) answered “No” to the question, “Is your walking speed faster than the speed of those of your age and sex?” The sustained group had lower odds of slow walking speed at 1 year (OR 0.60, 95% CI 0.38-0.95; *P*=.03), 2 years (OR 0.35, 95% CI 0.22-0.57; *P*<.001), and 3 years (OR 0.40, 95% CI 0.23-0.67; *P*=.001). Although not shown in the main table, the sustained group generally showed more favorable prevalence patterns for skipping breakfast, late-night dinner, and sufficient restful sleep than the comparison group; however, the differences were not consistent across follow-up years.

**Table 3.** Association between sustained self-reported behavior change stage and lifestyle items at 1, 2, and 3 years^a^.

Item year	Comparison, n/N (%)	Sustained, n/N (%)	Adjusted OR^b^ (95% CI)	*P* value
Exercise habit				
Year 1	74/368 (20.1)	116/229 (50.7)	3.40 (2.24-5.16)	<.001
Year 2	34/281 (12.1)	120/217 (55.3)	8.90 (5.26-15.07)	<.001
Year 3	30/219 (13.7)	93/171 (54.4)	6.06 (3.52-10.41)	<.001
Daily physical activity				
Year 1	47/367 (12.8)	71/229 (31.0)	2.51 (1.61-3.91)	<.001
Year 2	21/281 (7.5)	71/217 (32.7)	5.17 (2.93-9.13)	<.001
Year 3	18/218 (8.3)	50/171 (29.2)	4.08 (2.13-7.83)	<.001
Slow walking speed				
Year 1	249/368 (67.7)	121/227 (53.3)	0.60 (0.38-0.95)	.03
Year 2	197/282 (69.9)	104/216 (48.1)	0.35 (0.22-0.57)	<.001
Year 3	147/219 (67.1)	79/171 (46.2)	0.40 (0.23-0.67)	.001
Current smoking habit				
Year 1	123/390 (31.5)	63/247 (25.5)	0.36 (0.14-0.95)	.04
Year 2	95/299 (31.8)	56/233 (24.0)	0.32 (0.12-0.87)	.03
Year 3	74/228 (32.5)	42/184 (22.8)	0.26 (0.09-0.75)	.01

a n/N indicates the number of participants endorsing each lifestyle item among those with available data. Year indicates the years after time 0. Adjusted ORs compare the sustained group with the comparison group for endorsing each lifestyle item and were calculated using logistic regression models adjusted for baseline habit status, baseline age, and medication status for antihypertensive, antidiabetic, and lipid-lowering medications at both baseline and at each follow-up point.

b OR: odds ratio.

## Discussion

### Principal Findings

This retrospective cohort study examined the duration of maintenance of the self-reported behavior change stage and its associations with physiological markers and lifestyle behaviors among male bus drivers. First, maintenance typically began in midlife (mean age 47.2, SD 7.0 y) but was difficult to sustain, with a median retention time of 362 (IQR 0-1087) days. Second, participants who sustained the action or maintenance stage for at least 1.5 years showed more favorable physical activity–related lifestyle patterns and lower smoking prevalence. Third, sustained maintenance was associated with more favorable metabolic profiles, including lower BMI and waist circumference at 2 and 3 years, and higher HDL-C, lower TG, and slightly lower HbA_1c_ at 3 years, although these differences did not indicate complete normalization of metabolic risk.

### Self-Reported Behavior Change Stage Maintenance Emerged Around Midlife but Was Difficult to Sustain

An important finding of this study was that the maintenance episode of the self-reported behavior change stage among male bus drivers began in midlife. At time 0, the mean age was 47.24 (SD 6.78) years in the sustained group and 47.19 (SD 7.14) years in the comparison group. These nearly identical mean ages indicate little difference between the groups in the age at which the self-reported behavior change stage was initiated.

The initiation of the self-reported behavior change stage maintenance around the age of 47 years, as observed in this study, may be related to the occupational characteristics of bus drivers. Commercial motor vehicle drivers are an occupational group whose health status is routinely evaluated to ensure safe driving. In addition to regular health checkups, they undergo preduty and postduty roll calls, during which illness, fatigue, sleep deprivation, and other conditions are checked [7]. Moreover, because health status may affect driving eligibility and employment, emerging health risks may be perceived as practical concerns related to occupational life and income. Such institutional and organizational health management environments may therefore trigger health behavior change.

At the same time, such behavior changes may be initiated in response to health anxiety, workplace guidance, a sense of responsibility for continuing to drive, or a desire to avoid occupational disadvantages. In this case, motivation may be closer to external regulation or controlled motivation, as described in self-determination theory [16,17]. Although such motivation may contribute to the initiation of behavior change, it may be insufficient for long-term maintenance unless the behavior is internalized as part of an individual’s values and daily life [18]. In this study, the retention probability decreased to 57.7% at 6 months and 44.1% at 1 year after initiation of the self-reported behavior change stage, highlighting the difficulty of sustaining behavior change as part of daily life and work routines.

Therefore, health guidance for bus drivers should extend beyond communicating health checkup results or driving safety risks. It should help drivers view behavior change as a sustainable and worthwhile part of their efforts, rather than a temporary response to health concerns. Given the structural constraints specific to bus drivers, including early-morning and late-night shifts, irregular breaks, prolonged sitting, and restrictions on meal timing [19], support should include small, feasible behavioral goals that can be implemented during work and opportunities to accumulate experiences of successful continuation. Occupational health and nursing professionals play an important role in integrating health management that supports driving safety with support that promotes autonomous long-term behavior maintenance.

### Long-Term Maintenance of the Self-Reported Behavior Change Stage Was Associated With Physical Activity and More Favorable Metabolic Profiles

The second important finding was that participants who maintained the self-reported behavior change stage over the long term consistently showed more favorable physical activity–related lifestyle patterns and trends in selected physiological markers (BMI, WC, HDL-C, TG, and HbA_1c_). In the sustained group, approximately half of the participants reported having a regular exercise habit at 1, 2, and 3 years, whereas the proportion in the comparison group remained approximately 20%, even at its highest point. The proportion of participants who walked or engaged in an equivalent amount of physical activity for at least 1 hour per day in daily life was also consistently higher in the sustained group. In addition, the proportion of participants who answered that their walking speed was not faster than that of people of the same age and sex was significantly lower in the sustained group. These findings suggest that the self-reported behavior change stage in the sustained group may not have represented only intention or temporary efforts but may have been at least partly established as a habit of moving the body in daily life.

Regarding physiological markers, the sustained group had a significantly lower BMI and WC than the comparison group at 2 and 3 years. However, BMI in the sustained group remained within the 24 kg/m² range, suggesting that the findings should be interpreted not as a large reduction in body weight but rather as a suppression of the gradual increase in BMI observed in the comparison group. Similarly, although WC was lower in the sustained group than in the comparison group, the mean value did not fall below 85 cm throughout the follow-up period. Therefore, long-term maintenance of the self-reported behavior change stage may have been associated with the prevention of weight gain and increases in WC, rather than the complete resolution of obesity or visceral fat accumulation.

For lipid markers, HDL-C was consistently higher in the sustained group than in the comparison group from 1 to 3 years, and TG was significantly lower at 2 and 3 years. HbA_1c_ levels were also significantly lower in the sustained group at 3 years. However, these differences should be interpreted as the maintenance of a more favorable metabolic profile in the sustained group rather than the normalization of abnormal values or a large clinical improvement. In particular, the between-group difference in HbA_1c_ was small, and the adjusted mean values in both groups remained above the predefined at-risk threshold of 5.6%. Therefore, findings related to glycemic status require cautious interpretation.

Among lifestyle factors, current smoking also showed a more favorable pattern in the sustained group. The prevalence of current smoking in the sustained group decreased from 25.5% at 1 year to 22.8% at 3 years, whereas it increased slightly from 31.5% to 32.5% in the comparison group. According to a fiscal year 2023 survey of Specific Health Checkup questionnaire responses conducted by the National Federation of Health Insurance Societies [20], the proportion of men who reported habitual smoking was 31.4%, and the corresponding proportion among men aged 45 to 49 years—which is close to the mean age of the present study population—was 34.0%. The smoking prevalence in the comparison group in the present study was close to the national male average, whereas the sustained group showed a lower prevalence than both the national average and that among men of similar ages. However, because this study did not capture the timing of smoking initiation or cessation in detail, we cannot conclude that maintenance of the self-reported behavior change stage promotes smoking cessation. Rather, a health orientation toward nonsmoking or smoking cessation, self-management awareness, or sensitivity to health risks may be associated with long-term self-reported behavior change stage maintenance.

In contrast, challenges related to meal timing and sleep persisted even in the sustained group. In the same survey by the National Federation of Health Insurance Societies [20], 24.4% of men reported skipping breakfast at least 3 times per week, 37.9% reported eating dinner within 2 hours before bedtime at least 3 times per week, and 63.9% reported getting sufficient rest from sleep. Among men aged 45 to 49 years, the corresponding proportions were 29.6% for skipping breakfast, 42.9% for having late-night dinner, and 63.8% for having sufficient restful sleep. In contrast, in the present study, the prevalence of skipping breakfast was approximately 34% to 35% in the sustained group and 41% to 43% in the comparison group. The prevalence of late-night dinners was also high, at approximately 54% to 59% in the sustained group and 63% to 69% in the comparison group. In addition, the proportion of participants reporting sufficient restful sleep remained at approximately 46% to 54% in the sustained group and 38% to 44% in the comparison group. Although a direct comparison is limited by differences in age structure, occupation, and insurer composition, these findings suggest that male bus drivers may continue to face lifestyle challenges related to meal timing and restful sleep even when they maintain the self-reported behavior change stage over the long term.

Overall, long-term maintenance of the self-reported behavior change stage was mainly associated with established physical activity habits, lower smoking prevalence, the suppression of increases in BMI and WC, higher HDL-C levels, lower TG levels, and lower HbA_1c_ at 3 years. In contrast, lifestyle items related to daily rhythms, such as skipping breakfast, eating a late-night dinner, and having insufficient restful sleep, tended to be less favorable than those of men of similar age in the national survey, even among participants in the sustained group. These findings suggest that self-reported behavior change stage maintenance among bus drivers may be more readily reflected in individual behaviors, such as physical activity and smoking, whereas improvement in eating and sleeping habits may be limited by work schedules and driving timetables.

### Reconsidering Health Guidance Strategies: Evaluating Maintenance That Prevents Worsening, Not Only Numerical Improvement

The findings of this study suggest the need to broaden the evaluation framework for health guidance among bus drivers from focusing on numerical improvement alone to the maintenance of behaviors and prevention of risk progression. In the sustained group, physical activity–related lifestyle patterns were more favorable, BMI and WC were lower, HDL-C was higher, and TG and HbA_1c_ showed more favorable trends than in the comparison group. However, these differences did not indicate complete normalization of physiological risk. For example, the mean WC in the sustained group did not fall below 85 cm, and the mean HbA_1c_ levels in both groups remained above the at-risk status threshold of 5.6%. Therefore, the significance of these findings lies not in complete normalization but in their association with more favorable maintenance of weight, WC, lipid metabolism, and glycemic markers under aging-related and occupational constraints.

Bus drivers work in environments that may worsen metabolic risk, including prolonged sitting, irregular shifts, restrictions on meal timing, and disrupted sleep rhythms [19]. Therefore, even when marked weight reduction or normalization of laboratory values is not observed, maintaining health-related behaviors and preventing deterioration should be regarded as important outcomes in this occupational group. Because body weight and metabolic risk tend to increase with age during adulthood [21], the absence of large numerical improvements should not necessarily be interpreted as a failure.

Health guidance in Japan often emphasizes numerical improvement, which is useful for motivating risk reduction. However, when behavior change does not lead to visible numerical improvement, participants may feel that their efforts are wasted, potentially leading to resignation or resistance [22,23]. Repeated guidance without clear improvement may also reduce the effectiveness of support among repeat participants [24,25]. To address this issue, health guidance should incorporate process evaluation in addition to outcome evaluation. Professionals should provide feedback that frames the maintenance of feasible habits as a meaningful success. Such feedback may strengthen self-efficacy through mastery experience [26], and motivational interviewing may help affirm self-determined behaviors even when numerical indicators remain unchanged [27]. This approach is also consistent with self-determination theory, which emphasizes competence, autonomy, and more autonomous motivation [16,17].

Lin et al [12] reported that psychological empowerment and hedonic well-being, rather than information provision or inspiration alone, supported sustained behavior change among users of a smoking cessation app [12]. This perspective is relevant to bus drivers, for whom health checkup results, preduty and postduty roll calls, workplace guidance, and concerns about driving eligibility may serve as external triggers for behavior change. However, motivation based mainly on external pressure or crisis awareness may be insufficient for long-term maintenance. Health guidance should therefore foster psychological empowerment and well-being by helping drivers recognize small, feasible achievements within their work routines.

Similarly, digital and mHealth tools should not merely provide alerts or reminders. They should offer personalized, work routine–compatible goals, progress tracking, visualization of small achievements, and positive feedback on continued efforts. Examples include walking during breaks, reducing smoking, adjusting meals after late-night shifts, and finding ways to secure sleep. Such support may help transform health checkup results into feasible and meaningful actions that can be maintained under occupational constraints.

### Limitations and Future Directions

This study has several limitations. First, because this was a retrospective observational study, causal relationships between maintenance of the self-reported behavior change stage and changes in physiological markers could not be determined. The sustained and comparison groups were defined retrospectively according to the duration of maintenance after time 0; therefore, between-group differences should be interpreted as associations with long-term maintenance, not as effects of sustained behavior change.

Second, we analyzed each participant’s longest self-reported behavior change stage maintenance episode rather than the first episode. This approach was used to capture the most stable maintenance state observed during the study period, but it may have overestimated maintenance duration. Accordingly, the Kaplan-Meier estimates should be interpreted as the distribution of the longest maintenance episode, not as the probability of continuation after the initial behavior change.

Third, behavior change stage and lifestyle habits were assessed using SAQs and may have been affected by recall and social desirability biases. In addition, the behavior change stage item captured self-reported intention and engagement in lifestyle improvement, not objective behavioral quantities such as physical activity, dietary intake, sleep duration, or amount of smoking.

Fourth, initiation and discontinuation of the self-reported behavior change stage could be identified only during health checkups. Therefore, the actual timing of these transitions may have occurred between visits, and interval censoring may have affected the estimates of maintenance duration. Differences in checkup frequency over time, with annual checkups before 2014 and semiannual checkups from 2014 onward, may also have influenced duration estimates.

Fifth, the study population was limited to male bus drivers from a single company in Japan, which may limit generalizability. Although medication use was adjusted for at baseline and at follow-up, detailed information on medication type, dose, adherence, and treatment changes was not available. In addition, psychological, social, and occupational factors that may influence behavior change maintenance, such as self-efficacy, health literacy, workplace support, work shifts, break availability, and anxiety about continuing to drive, were not fully measured.

Future studies should use prospective designs to follow the process of initiation, maintenance, discontinuation, and resumption of behavior change in greater detail. They should also incorporate objective measures of physical activity, sedentary time, sleep, and meal timing using wearable devices or smartphone applications, together with psychosocial and occupational factors. Qualitative studies may also help identify the strategies used by drivers who maintain behavior change and the barriers experienced by those who discontinue it. Such evidence may support the development of individualized occupational health guidance models that consider not only medical risk but also psychological readiness and the work environment.

### Conclusions

Using longitudinal occupational health checkup data from male bus drivers, this study showed that the maintenance of the self-reported behavior change stage often began in midlife but was difficult to sustain beyond 1 year. Long-term maintenance was associated with more favorable physical activity–related lifestyle patterns, lower smoking prevalence, and more favorable metabolic profiles, although these findings did not indicate complete normalization of metabolic risk. These results suggest that occupational health guidance for bus drivers should evaluate not only numerical improvements but also sustained behaviors and the prevention of worsening under occupational constraints. Tailored long-term support that addresses work schedules, meal timing, breaks, physical activity, smoking, and sleep rhythms may be needed to help drivers maintain feasible health behaviors over time.

## Supplementary material

10.2196/93645 Multimedia Appendix 1 Definitions of questionnaire items and self-reported behavior change stage, and supplementary results of physiological marker and lifestyle item analyses across all follow-up points.

## Abbreviations

ANCOVA: analysis of covariance
BP: blood pressure
DBP: diastolic blood pressure
HbA_1c_: glycated hemoglobin
HDL-C: high-density lipoprotein cholesterol
LDL-C: low-density lipoprotein cholesterol
mHealth: mobile health
NGSP: National Glycohemoglobin Standardization Program
OR: odds ratio
SAQ: Standard Self-administered Questionnaire
SBP: systolic blood pressure
TG: triglycerides
WC: waist circumference
