# Supplementary material for: Maintenance of the Self-Reported Behavior Change Stage Among Male Bus Drivers Using Longitudinal Occupational Health Checkup Data: Retrospective Cohort Study
Source: Asian Pac Isl Nurs J. 2026 Sep 1;10:e93645. doi: 10.2196/93645 (PMC13533414; doi:10.2196/93645)
Supplement: Supplementary file 1 [file apinj-v10-e93645-s001.docx]

**Table S1. Definitions of lifestyle habits and medication status derived from the Standard Questionnaires for Specific Health Checkups** [14]**.**

| Category | Variable | Questionnaire Item / Definition | Criteria for Positive Status |
| --- | --- | --- | --- |
| Medication  history | antihypertensive drug | Are you taking medication to reduce blood pressure? | Answered "Yes" |
|  | antidiabetic drug | Are you taking medication to reduce blood sugar or insulin injection? | Answered "Yes" |
|  | lipid-lowering drug | Are you taking medication to reduce your level of cholesterol or of neutral fat? | Answered "Yes" |
| Physical Activity | Exercise habit | Are you in a habit of doing exercise to sweat lightly for over 30 minutes at a time, 2 times weekly, for over a year? | Answered "Yes" |
|  | Daily physical activity | In your daily life do you walk or do any equivalent amount of physical activity for more than one hour a day? | Answered "Yes" |
|  | Slow or non-fast walking speed | Is your walking speed faster than the speed of those of your age and sex? | Answered "No" |
| Dietary and Other Habits | Eating quickly | Is your eating speed quicker than others? | Answered "Quicker" |
|  | Late-night dinner | Do you eat supper two hours before bedtime more than 3 times a week? | Answered "Yes" |
|  | Skipping breakfast | Do you skip breakfast more than 3 times a week? | Answered "Yes" |
|  | Daily alcohol consumption | How often do you drink? | Answered "Every day" |
|  | Current smoking habit | Are you currently a smoker? (Defined as those who have smoked a total of over 100 cigarettes or have smoked over a period of 6 months and have been smoking over the past month). | Answered "Yes" |
|  | Sufficient restful sleep | Do you sleep well and enough? | Answered "Yes" |
|  |  |  |  |

**Table S2. Operational definitions of Self-Reported Behavior Change Stage** [14]**.**

| Variable | Question Item | Response Options (Self-reported) | Study Classification |
| --- | --- | --- | --- |
| Self-Reported Behavior Change | Do you intend to improve your eating and exercise habits? | (1) No intention to improve.  (2) Intend to improve within the next 6 months.  (3) Intend to improve within the next month and have already started taking action.  (4) Already taking action (for less than 6 months).  (5) Already taking action (for 6 months or more). | Pre-action/preparatory stages  Answered (1) to (3)  Coded as 0  Action/maintenance stages  Answered (4) or (5)  Coded as 1 |

**Table S3. ANCOVA-Adjusted Comparison of Physiological Markers Over 3 Years**

| **Item / Year** | **n** | **Crude** |  | **Adjusted** |  | **Difference (95% CI)** | ***P* value** |
| --- | --- | --- | --- | --- | --- | --- | --- |
|  |  | **Comparison** | **Sustained** | **Comparison** | **Sustained** |  |  |
| **Body mass index (kg/m²)** | | |  |  |  |  |  |
| 0.5 years | 338 / 187 | 25.10 (4.35) | 24.30 (3.33) | 24.81 | 24.82 | 0.01 (-0.12 to 0.14) | .84 |
| 1.0 years | 390 / 247 | 25.10 (4.07) | 24.46 (3.58) | 24.89 | 24.78 | -0.12 (-0.26 to 0.03) | .11 |
| 1.5 years | 264 / 202 | 25.15 (4.28) | 24.30 (3.39) | 24.83 | 24.72 | -0.11 (-0.29 to 0.08) | .26 |
| 2.0 years | 299 / 233 | 25.22 (3.92) | 24.37 (3.54) | 25.01 | 24.64 | -0.37 (-0.57 to -0.17) | <.001 |
| 2.5 years | 202 / 178 | 25.19 (3.94) | 24.24 (3.43) | 24.86 | 24.62 | -0.24 (-0.50 to 0.01) | .06 |
| 3.0 years | 228 / 184 | 25.42 (3.84) | 24.58 (3.69) | 25.21 | 24.85 | -0.36 (-0.61 to -0.10) | .006 |
| **Waist circumference (cm)** | | | |  |  |  |  |
| 0.5 years | 338 / 187 | 88.00 (11.46) | 85.80 (8.91) | 87.24 | 87.19 | -0.05 (-0.52 to 0.42) | .84 |
| 1.0 years | 390 / 247 | 87.81 (10.94) | 85.74 (9.46) | 87.11 | 86.83 | -0.28 (-0.80 to 0.24) | .29 |
| 1.5 years | 264 / 202 | 88.32 (11.24) | 85.98 (8.93) | 87.39 | 87.2 | -0.18 (-0.83 to 0.46) | .57 |
| 2.0 years | 299 / 233 | 88.30 (10.27) | 85.29 (9.27) | 87.54 | 86.27 | -1.27 (-1.93 to -0.60) | <.001 |
| 2.5 years | 202 / 178 | 88.43 (10.23) | 85.47 (9.13) | 87.33 | 86.71 | -0.62 (-1.48 to 0.24) | .16 |
| 3.0 years | 228 / 184 | 89.06 (10.17) | 85.73 (9.59) | 88.13 | 86.89 | -1.24 (-2.10 to -0.38) | .005 |
| **Systolic blood pressure (mm Hg)** | | | |  |  |  |  |
| 0.5 years | 338 / 187 | 128.91 (13.04) | 127.42 (11.40) | 128.76 | 127.69 | -1.07 (-2.87 to 0.73) | .24 |
| 1.0 years | 390 / 247 | 129.51 (12.85) | 129.08 (12.81) | 129.39 | 129.27 | -0.12 (-1.86 to 1.61) | .89 |
| 1.5 years | 264 / 202 | 129.94 (12.62) | 129.34 (12.25) | 129.74 | 129.59 | -0.16 (-2.11 to 1.80) | .87 |
| 2.0 years | 299 / 233 | 129.20 (12.15) | 129.96 (14.52) | 129.01 | 130.21 | 1.21 (-0.81 to 3.22) | .24 |
| 2.5 years | 202 / 178 | 129.05 (12.62) | 129.25 (11.79) | 129.15 | 129.14 | -0.01 (-2.17 to 2.15) | .99 |
| 3.0 years | 228 / 184 | 129.47 (12.75) | 128.64 (12.65) | 129.35 | 128.79 | -0.56 (-2.79 to 1.67) | .62 |
| **Diastolic blood pressure (mm Hg)** | | | |  |  |  |  |
| 0.5 years | 338 / 187 | 83.01 (9.78) | 81.30 (9.86) | 82.84 | 81.61 | -1.23 (-2.48 to 0.02) | .053 |
| 1.0 years | 390 / 247 | 83.21 (10.10) | 82.82 (9.84) | 83 | 83.15 | 0.15 (-1.06 to 1.37) | .80 |
| 1.5 years | 264 / 202 | 83.91 (10.33) | 82.87 (9.63) | 83.87 | 82.92 | -0.95 (-2.38 to 0.48) | .19 |
| 2.0 years | 299 / 233 | 84.02 (9.82) | 83.44 (9.87) | 83.72 | 83.82 | 0.10 (-1.27 to 1.47) | .89 |
| 2.5 years | 202 / 178 | 84.87 (9.87) | 83.56 (9.86) | 84.84 | 83.58 | -1.26 (-2.84 to 0.32) | .12 |
| 3.0 years | 228 / 184 | 84.31 (9.75) | 82.82 (8.91) | 84.03 | 83.16 | -0.87 (-2.43 to 0.69) | .28 |
| **Hemoglobin A1c (%)** | | |  |  |  |  |  |
| 0.5 years | 338 / 187 | 5.72 (0.55) | 5.64 (0.49) | 5.70 | 5.69 | -0.01 (-0.04 to 0.03) | .74 |
| 1.0 years | 390 / 247 | 5.70 (0.60) | 5.70 (0.58) | 5.70 | 5.69 | -0.01 (-0.07 to 0.05) | .74 |
| 1.5 years | 264 / 202 | 5.75 (0.53) | 5.70 (0.57) | 5.74 | 5.72 | -0.02 (-0.07 to 0.04) | .55 |
| 2.0 years | 299 / 233 | 5.72 (0.60) | 5.71 (0.59) | 5.73 | 5.70 | -0.03 (-0.08 to 0.01) | .17 |
| 2.5 years | 202 / 178 | 5.80 (0.63) | 5.68 (0.47) | 5.76 | 5.71 | -0.05 (-0.11 to 0.01) | .08 |
| 3.0 years | 228 / 184 | 5.80 (0.61) | 5.72 (0.55) | 5.79 | 5.73 | -0.07 (-0.12 to -0.01) | .02 |
| **Triglycerides (mg/dL)** | | | |  |  |  |  |
| 0.5 years | 338 / 187 | 136.73 (115.19) | 132.01 (88.49) | 133.65 | 137.59 | 3.94 (-10.87 to 18.75) | .60 |
| 1.0 years | 390 / 247 | 132.98 (98.14) | 134.53 (93.61) | 131.08 | 137.52 | 6.44 (-6.63 to 19.51) | .33 |
| 1.5 years | 264 / 202 | 137.09 (85.25) | 133.34 (94.80) | 133.89 | 137.53 | 3.64 (-9.34 to 16.61) | .58 |
| 2.0 years | 299 / 233 | 145.88 (97.71) | 123.25 (83.15) | 143.43 | 126.4 | -17.03 (-30.51 to -3.56) | .01 |
| 2.5 years | 202 / 178 | 143.94 (86.82) | 131.81 (126.04) | 139.32 | 137.04 | -2.28 (-20.32 to 15.76) | .80 |
| 3.0 years | 228 / 184 | 144.62 (88.86) | 118.46 (87.92) | 142.41 | 121.2 | -21.22 (-37.28 to -5.15) | .01 |
| **High-density lipoprotein cholesterol (mg/dL)** | | | |  |  |  |  |
| 0.5 years | 338 / 187 | 58.23 (15.57) | 59.34 (14.70) | 58.48 | 58.88 | 0.40 (-0.82 to 1.62) | .52 |
| 1.0 years | 390 / 247 | 58.16 (15.73) | 60.70 (15.81) | 58.63 | 59.94 | 1.31 (0.11 to 2.50) | .03 |
| 1.5 years | 264 / 202 | 56.14 (14.64) | 59.36 (15.48) | 56.62 | 58.74 | 2.12 (0.80 to 3.44) | .002 |
| 2.0 years | 299 / 233 | 56.76 (16.06) | 61.46 (16.56) | 57.43 | 60.6 | 3.17 (1.80 to 4.53) | <.001 |
| 2.5 years | 202 / 178 | 55.85 (15.21) | 60.03 (15.61) | 56.96 | 58.76 | 1.80 (0.16 to 3.44) | .03 |
| 3.0 years | 228 / 184 | 56.00 (14.42) | 61.97 (16.28) | 57.02 | 60.71 | 3.69 (2.09 to 5.29) | <.001 |
| **Low-density lipoprotein cholesterol (mg/dL)** | | | |  |  |  |  |
| 0.5 years | 338 / 187 | 120.98 (27.04) | 123.92 (25.51) | 121.42 | 123.12 | 1.70 (-1.67 to 5.08) | .32 |
| 1.0 years | 390 / 247 | 126.65 (27.35) | 126.46 (26.66) | 126.8 | 126.22 | -0.58 (-3.71 to 2.56) | .72 |
| 1.5 years | 264 / 202 | 118.60 (26.88) | 124.16 (26.14) | 118.37 | 124.46 | 6.09 (2.23 to 9.95) | .002 |
| 2.0 years | 299 / 233 | 128.29 (27.10) | 127.09 (28.89) | 127.94 | 127.54 | -0.40 (-4.22 to 3.42) | .84 |
| 2.5 years | 202 / 178 | 120.66 (26.24) | 121.50 (26.93) | 120.93 | 121.19 | 0.26 (-4.11 to 4.63) | .91 |
| 3.0 years | 228 / 184 | 127.63 (24.97) | 126.01 (25.42) | 127.15 | 126.6 | -0.55 (-4.61 to 3.51) | .79 |

Note: Values in the n column are presented as comparison group/sustained group. Crude values are presented as mean (SD). Adjusted values are adjusted means estimated using analysis of covariance. Difference indicates the adjusted mean difference, calculated as the sustained group minus the comparison group. Negative values indicate lower adjusted means in the sustained group than in the comparison group. Differences, 95% CIs, and P values were calculated using analysis of covariance, adjusting for the baseline value of each respective marker, baseline age, and medication status for antihypertensive, antidiabetic, and lipid-lowering medications at both baseline and each follow-up point.

CI: confidence interval.

**Table S4. Detailed Results of Longitudinal Logistic Regression Analysis for Lifestyle Habits Across Follow-Up Time Points (0.5 to 3.0 Years)**

| **Item / Year** | **Comparison** | **Sustained** | **Adjusted OR (95% CI)** | ***P* value** |
| --- | --- | --- | --- | --- |
| **Exercise habit** | |  |  |  |
| 0.5 years | 33/212 (15.6%) | 46/110 (41.8%) | 2.85 (1.53 to 5.31) | .001 |
| 1.0 years | 74/368 (20.1%) | 116/229 (50.7%) | 3.40 (2.24 to 5.16) | <.001 |
| 1.5 years | 25/165 (15.2%) | 77/142 (54.2%) | 6.79 (3.45 to 13.36) | <.001 |
| 2.0 years | 34/281 (12.1%) | 120/217 (55.3%) | 8.90 (5.26 to 15.07) | <.001 |
| 2.5 years | 17/132 (12.9%) | 66/117 (56.4%) | 6.44 (3.07 to 13.52) | <.001 |
| 3.0 years | 30/219 (13.7%) | 93/171 (54.4%) | 6.06 (3.52 to 10.41) | <.001 |
| **Daily physical activity** | |  |  |  |
| 0.5 years | 22/212 (10.4%) | 30/110 (27.3%) | 2.81 (1.39 to 5.68) | .004 |
| 1.0 years | 47/367 (12.8%) | 71/229 (31.0%) | 2.51 (1.61 to 3.91) | <.001 |
| 1.5 years | 19/164 (11.6%) | 46/139 (33.1%) | 3.47 (1.76 to 6.85) | <.001 |
| 2.0 years | 21/281 (7.5%) | 71/217 (32.7%) | 5.17 (2.93 to 9.13) | <.001 |
| 2.5 years | 7/132 (5.3%) | 33/115 (28.7%) | 6.20 (2.36 to 16.28) | <.001 |
| 3.0 years | 18/218 (8.3%) | 50/171 (29.2%) | 4.08 (2.13 to 7.83) | <.001 |
| **Slow walking speed** | |  |  |  |
| 0.5 years | 146/213 (68.5%) | 63/109 (57.8%) | 0.69 (0.37 to 1.28) | .24 |
| 1.0 years | 249/368 (67.7%) | 121/227 (53.3%) | 0.60 (0.38 to 0.95) | .03 |
| 1.5 years | 114/164 (69.5%) | 71/139 (51.1%) | 0.41 (0.21 to 0.79) | .008 |
| 2.0 years | 197/282 (69.9%) | 104/216 (48.1%) | 0.35 (0.22 to 0.57) | <.001 |
| 2.5 years | 97/132 (73.5%) | 56/116 (48.3%) | 0.31 (0.16 to 0.62) | .001 |
| 3.0 years | 147/219 (67.1%) | 79/171 (46.2%) | 0.40 (0.23 to 0.67) | .001 |
| **Eating quickly** | |  |  |  |
| 0.5 years | 87/212 (41.0%) | 36/109 (33.0%) | 0.76 (0.37 to 1.55) | .45 |
| 1.0 years | 129/367 (35.1%) | 82/229 (35.8%) | 1.28 (0.78 to 2.10) | .33 |
| 1.5 years | 73/164 (44.5%) | 55/142 (38.7%) | 0.85 (0.43 to 1.69) | .64 |
| 2.0 years | 97/282 (34.4%) | 72/218 (33.0%) | 0.96 (0.58 to 1.59) | .88 |
| 2.5 years | 57/132 (43.2%) | 42/119 (35.3%) | 0.41 (0.17 to 0.99) | .048 |
| 3.0 years | 77/219 (35.2%) | 51/171 (29.8%) | 0.75 (0.41 to 1.37) | .35 |
| **Late-night dinner** | |  |  |  |
| 0.5 years | 131/205 (63.9%) | 67/109 (61.5%) | 1.46 (0.79 to 2.71) | .23 |
| 1.0 years | 238/364 (65.4%) | 134/228 (58.8%) | 0.89 (0.60 to 1.32) | .56 |
| 1.5 years | N/A | N/A | — | — |
| 2.0 years | 192/278 (69.1%) | 117/216 (54.2%) | 0.59 (0.38 to 0.91) | .02 |
| 2.5 years | 82/126 (65.1%) | 70/118 (59.3%) | 0.98 (0.52 to 1.86) | .96 |
| 3.0 years | 136/215 (63.3%) | 100/169 (59.2%) | 1.16 (0.70 to 1.90) | .57 |
| **Skipping breakfast** | |  |  |  |
| 0.5 years | 95/206 (46.1%) | 33/110 (30.0%) | 0.33 (0.15 to 0.74) | .007 |
| 1.0 years | 151/365 (41.4%) | 79/226 (35.0%) | 0.81 (0.50 to 1.30) | .39 |
| 1.5 years | 72/161 (44.7%) | 49/142 (34.5%) | 0.49 (0.23 to 1.01) | .054 |
| 2.0 years | 116/280 (41.4%) | 75/218 (34.4%) | 0.97 (0.58 to 1.64) | .92 |
| 2.5 years | 66/133 (49.6%) | 44/116 (37.9%) | 0.62 (0.32 to 1.22) | .17 |
| 3.0 years | 94/218 (43.1%) | 58/168 (34.5%) | 0.89 (0.52 to 1.54) | .69 |
| **Drinking alcohol every day** | |  |  |  |
| 0.5 years | 30/213 (14.1%) | 11/110 (10.0%) | 1.92 (0.42 to 8.86) | .40 |
| 1.0 years | 53/368 (14.4%) | 29/229 (12.7%) | 1.11 (0.49 to 2.48) | .80 |
| 1.5 years | 25/163 (15.3%) | 14/142 (9.9%) | 0.64 (0.21 to 1.93) | .43 |
| 2.0 years | 43/282 (15.2%) | 25/218 (11.5%) | 0.67 (0.31 to 1.46) | .32 |
| 2.5 years | 21/131 (16.0%) | 16/118 (13.6%) | 1.07 (0.32 to 3.57) | .92 |
| 3.0 years | 36/219 (16.4%) | 25/171 (14.6%) | 0.88 (0.40 to 1.93) | .75 |
| **Current smoking habit** | |  |  |  |
| 0.5 years | 102/338 (30.2%) | 49/187 (26.2%) | 0.99 (0.22 to 4.53) | .99 |
| 1.0 years | 123/390 (31.5%) | 63/247 (25.5%) | 0.36 (0.14 to 0.95) | .04 |
| 1.5 years | 89/263 (33.8%) | 49/202 (24.3%) | 0.15 (0.04 to 0.54) | .004 |
| 2.0 years | 95/299 (31.8%) | 56/233 (24.0%) | 0.32 (0.12 to 0.87) | .03 |
| 2.5 years | 65/202 (32.2%) | 41/177 (23.2%) | 0.27 (0.09 to 0.77) | .02 |
| 3.0 years | 74/228 (32.5%) | 42/184 (22.8%) | 0.26 (0.09 to 0.75) | .01 |
| **Sufficient restful sleep** | |  |  |  |
| 0.5 years | 95/200 (47.5%) | 53/103 (51.5%) | 2.70 (1.36 to 5.38) | .005 |
| 1.0 years | 154/361 (42.7%) | 113/221 (51.1%) | 1.41 (0.93 to 2.15) | .11 |
| 1.5 years | 67/158 (42.4%) | 59/131 (45.0%) | 1.63 (0.90 to 2.98) | .11 |
| 2.0 years | 120/275 (43.6%) | 115/215 (53.5%) | 1.28 (0.83 to 1.98) | .26 |
| 2.5 years | 51/124 (41.1%) | 50/109 (45.9%) | 1.44 (0.75 to 2.76) | .27 |
| 3.0 years | 82/217 (37.8%) | 77/169 (45.6%) | 1.15 (0.69 to 1.90) | .60 |

Note: Values in the comparison and sustained columns are presented as n/N (%), where n indicates the number of participants meeting the positive status criterion for each lifestyle item and N indicates the number of participants with available data at each follow-up point. Year indicates years after Time 0. Adjusted ORs compare the sustained group with the comparison group for meeting the positive status criterion for each lifestyle item. ORs and 95% CIs were calculated using multivariable logistic regression models adjusted for baseline status of the corresponding lifestyle item, baseline age, and medication status for antihypertensive, antidiabetic, and lipid-lowering medications at both baseline and each follow-up point. The definitions of positive status for lifestyle items and medication status are provided in Table S1. N/A and em dashes indicate that data or model estimates were not available.

CI: confidence interval; OR: odds ratio.
